# Dynamic Shifts in Gut Microbiota and Metabolic Pathways of Xinggao Mutton Sheep During Weaning: A Multi-Omics Analysis

**DOI:** 10.3390/ani16101532

**Published:** 2026-05-16

**Authors:** Xiaolong He, Jingda Yuan, Biao Wang, Qing Qin, Rigele Te, Lai Da, Xuewen Liu, Shaoyin Fu

**Affiliations:** 1 Inner Mongolia Academy of Agricultural & Animal Husbandry Sciences, No. 22 Zhaojun Road, Hohhot 010031, China; hexiaolong1983@163.com (X.H.); yuanjingda0825@163.com (J.Y.); wangbnd@163.com (B.W.); steveqin_nmg@163.com (Q.Q.); terigele-2009@163.com (R.T.); dalai1023@163.com (L.D.); 2 National and Local Joint Engineering Research Center for Genetic Resource Evaluation and Breeding Technology of Mutton Sheep, Inner Mongolia Academy of Agricultural & Animal Husbandry Sciences, No. 22 Zhaojun Road, Hohhot 010031, China; 3 College of Animal Science, Xing’an Polytechnic University, No. 160 Wucha Road, Ulanhot 137700, China; xamlxw8718@126.com

**Keywords:** Xinggao mutton sheep, weaning, gut microorganisms, 16S rDNA amplicon sequencing, metabolomics

## Abstract

The composition and function of the intestinal microbiota of Xinggao mutton sheep were explored before and post-weaning using non-target metabolomics. Both 16S rDNA amplicon sequencing technology and serum non-target metabolomics were used to determine changes in sheep metabolites and intestinal flora. A random sampling method was used to collect fecal samples from 20 pre-weaning lambs and 20 lambs post-weaning. Analysis of the intestinal flora identified *Firmicutes* as the predominant phylum pre-weaning and *Bacteroidota* as the dominant phylum post-weaning. Joint multi-omics analysis revealed that *Oscillospiraceae UCG-005* and *Prevotella_9* were significantly correlated with serum L-tyrosine, Indole-3-acrylic acid, and taurine-related metabolites, suggesting their potential roles in modulating digestive health in Xinggao mutton sheep.

## Simple Summary

This study investigated intestinal microbial and serum metabolic changes, along with their correlations, in Xinggao mutton sheep pre- and post-weaning. The dominant intestinal phylum shifted from *Firmicutes* to *Bacteroidota* after weaning. Multi-omics analysis revealed associations between specific bacteria and metabolite production. These findings clarify the weaning adaptation mechanism and provide a theoretical basis for optimizing breeding strategies.

## 1. Introduction

The Xinggao Mutton Sheep is a newly cultivated mutton sheep breed in eastern Inner Mongolia, characterized by rapid growth, high reproductive efficiency, and excellent breeding performance. As typical ruminants, the intestinal tract of this sheep breed plays a pivotal role in diverse physiological processes, including feed digestion, nutrient absorption, and immune regulation. The intestine harbors a highly diverse microbial community that executes essential functions for the host, such as supplying microbial metabolites as nutritional substrates and participating in host immune homeostasis [1].

Weaning constitutes a highly stressful and critical developmental transition for young ruminants, frequently inducing pronounced alterations in gut microbial composition and metabolic profiles, thereby exerting adverse effects on animal health and productivity [2]. This transitional period is marked by abrupt dietary changes and multiple environmental stressors, which impose severe challenges on the immature digestive and immune systems of lambs [3]. The rumen microbiota of lambs undergoes gradual development and functional maturation with increasing age. The initial colonization of the rumen microbial community commences at birth, mainly via vertical microbial transmission from ewes, which represents a key pathway for establishing the gastrointestinal microbiome in neonatal ruminants [4]. Cellulolytic bacteria can be detected as early as 3–5 days of age in lambs and reach high abundance by 2–3 weeks of age [5]. Numerous studies have explored strategies targeting the early–life microbiota to modulate rumen fermentation, including dietary manipulation, optimized weaning regimes, rumen fluid inoculation, and feed supplementation [6,7,8].

Previous studies have demonstrated that *Prevotella* and *Firmicutes* are the dominant bacterial taxa in the stomach of various ruminant species, including cattle, buffalo, yaks, goats, sheep, and roe deer, whereas *Bacteroidetes*, *Clostridium*, *Alistipes*, and *Ruminococcus* predominate in the large intestine [9]. Gastrointestinal microbial metabolites are closely associated with complex host physiological processes, including immune function and inflammatory responses. Gut microbes not only assist the host in digesting and fermenting feed to generate metabolites but also synthesize a variety of bioactive compounds, such as secondary bile acids and polysaccharide A [10]. Extensive crosstalk occurs among these microbes, their metabolites, and the host’s innate and adaptive immune systems [11].

Although the general characteristics of the ruminant gastrointestinal microbiome have been widely documented, the specific developmental succession, compositional uniqueness, and functional maturation of the intestinal and rumen microbial communities in the newly cultivated Xinggao Mutton Sheep remain largely unexplored. The lack of breed-specific data creates a critical research gap, restricting the implementation of precision nutrition, early-life microbiome interventions, and microbiome-assisted genetic breeding strategies tailored to this high-performance sheep breed. Accordingly, the present study was designed to fill this knowledge gap by comprehensively characterizing the dynamic changes in the gut microbiota and serum metabolome of Xinggao Mutton Sheep before and post-weaning.

An integrated multi-omics approach combining 16S rRNA gene amplicon sequencing and untargeted metabolomics was applied. The findings of this study establish the first comprehensive map of the gut microbiota-metabolome axis for a mutton sheep breed during the weaning stage, providing important theoretical support for subsequent research and practical applications. This multi-omics framework extends beyond conventional microbial profiling and facilitates the development of translatable management strategies that effectively improve lamb welfare and production robustness while deepening the understanding of host-microbe interactions in agricultural livestock systems.

## 2. Materials and Methods

### 2.1. Experimental Animal

This study was conducted at the Inner Mongolia Academy of Agricultural and Animal Husbandry Sciences. The test animals were Xinggao meat sheep (collected from Jalaid, Xing’an League, Inner Mongolia Autonomous Region). All lambs selected in this study were female. A random sampling method was used to collect fecal and serum samples from 20 pre-weaning lambs (35–50 days of age) and 20 lambs one month post-weaning. These 40 lambs were divided into two groups: 20 in the lactation period (PW group, body weight of 16.88 ± 1.02 kg), which were fed both ewe’s milk and starter feed, and 20 in the post-weaning period (AW group, weaned at 60 days of age, body weight of 21.13 ± 0.70 kg). Fecal samples from the pre-weaning group (PW group) were collected at random time points within the 35–50-day age range, while all post-weaning group (AW group) samples were collected exactly one month after weaning.

During the experiment, the AW group lambs were housed together in a single pen (5 × 3 m, iron-fenced, with plastic slatted flooring) to better reflect real production conditions. Environmental temperature was continuously monitored throughout the experimental period to ensure that all lambs were raised under appropriate temperature conditions. The AW group lambs were fed the same diet at 9 a.m. and 5 p.m. daily, and all lambs had free access to water throughout the experiment. The ingredients and nutritional composition of the diets are presented in Supplementary Table S1.

### 2.2. Sample Collection

Before sampling, all lambs were examined by professionals to confirm they were in a healthy physiological state. During the collection process, manual compression was applied around the lamb’s anus, and fresh fecal samples were obtained from the rectum using disposable PE gloves (pre-lubricated). The samples were immediately placed in sterile, enzyme-free sampling tubes and stored on ice. Meanwhile, venous blood samples (5 mL per lamb) were collected from the jugular vein of each lamb into plain tubes (without additives, Model KJ030A) using sterile syringes. The blood samples were left at room temperature for 30 min to coagulate, then centrifuged at 3000 r/min and 4 °C for 10 min to separate the serum. The separated serum was transferred into sterile, cryopreservation tubes- and stored on ice together with the fecal samples.

Subsequently, both the fecal samples and serum samples were transported to the laboratory on dry ice. The fecal samples were stored in a −80 °C freezer for subsequent microbiome analysis, and the serum samples were stored in the same −80 °C freezer for subsequent metabolomics analysis (using serum metabolites).

### 2.3. 16S rDNA Amplicon Sequencing Technology

#### 2.3.1. DNA Extraction and Library Preparation

Total genomic DNA was extracted from fecal samples using the TIANamp Stool DNA Extraction Kit (Catalog No. DP328, TIANGEN, Beijing, China). The Universal DNA Purification and Recovery Kit (Catalog No. DP214–03, TIANGEN, Beijing, China) was used for DNA purification throughout the experimental procedure to guarantee qualified DNA templates for subsequent library preparation and sequencing.

#### 2.3.2. Experimental Procedure

The 16S rRNA V4 hypervariable region was amplified using primers 515F (5′-GTGCCAGCMGCCGCGGTAA-3′) and 806R (5′-GGACTACHVGGGTWTCTAAT-3′). Each PCR reaction contained 15 μL of Phusion^®^ High-Fidelity PCR Master Mix (New England Biolabs, Ipswich, MA, USA), 0.2 μM of each primer, and approximately 10 ng template DNA. Thermal cycling began with an initial denaturation at 98 °C for 1 min, followed by 30 cycles of 98 °C for 10 s, 50 °C for 30 s, and 72 °C for 30 s, with a final extension at 72 °C for 5 min. Amplicons were subsequently purified using magnetic beads, then pooled at equal density ratios according to their measured concentrations. Target bands were verified via electrophoresis and gel-recovered prior to library construction. Sequencing libraries were prepared with index addition, then quality-assessed via Qubit fluorometry and real-time PCR for quantification, alongside bioanalyzer profiling for size distribution. Finally, the quantified libraries were pooled and subjected to sequencing on Illumina platforms (Illumina Inc., San Diego, CA, USA) based on effective library concentration and required data output.

#### 2.3.3. Bioinformatic Analysis Pipeline

Paired-end reads were demultiplexed into samples according to their unique barcodes, followed by trimming of barcode and primer sequences. For sequence assembly, paired-end reads were merged with FLASH (V1.2.11, http://ccb.jhu.edu/software/FLASH/, accessed on July 2024) [12], an efficient and precise tool developed for merging paired-end reads in which a portion of reads overlaps with those derived from the opposite end of the same DNA fragment; the resulting merged sequences are designated raw tags. For data filtration, raw tags were subsequently quality-filtered using Fastp (Version 0.23.1) to generate high-quality clean tags [13]. The clean tags were then aligned against the Silva database (16S; https://www.arb-silva.de/, accessed on July 2024) for chimera detection, and chimeric sequences were eliminated using the vsearch package (V2.16.0, https://github.com/torognes/vsearch, accessed on July 2024) [14] to yield effective tags.

#### 2.3.4. ASV Denoising and Species Annotation

Effective tags were denoised using DADA2 within QIIME2 (Version QIIME2–202202) with parameters set as --p-trunc-len 0, --p-max-ee 2 and --p-pooling-method independent to generate amplicon sequence variants (ASVs). Species annotation was subsequently performed in QIIME2 against the NCBI NT database (https://www.ncbi.nlm.nih.gov/nucleotide/, accessed on July 2024), focusing on bacterial 16S rRNA sequences, to characterize the taxonomic composition of microbial communities across samples.

ASV absolute abundances were rarefied to 49,444 sequences per sample (the minimum sequence count observed across all 40 samples), and all downstream alpha and beta diversity analyses were conducted on the normalized dataset. At each taxonomic rank (phylum, class, order, family, genus, and species), the top 10 taxa per sample were selected to generate relative abundance distribution histograms, while abundance data for the top 35 taxa were used to construct heatmaps via the R pheatmap 1.0.12 function, enabling visual representation of abundance levels and taxa clustering. Venn diagrams were generated in R via the VennDiagram function.

#### 2.3.5. Alpha Diversity

To analyze the diversity, richness, and uniformity of the communities in the samples, alpha diversity was calculated using seven indices in QIIME2: Observed_ASVs, Chao1, Shannon, Simpson, Dominance, Good’s coverage, and Pielou_e [15]. Two indices were selected to characterize community richness: Observed_ASVs, representing the number of observed species, and Chao1, the Chao1 richness estimator [16]. Three indices were used to assess community diversity: the Shannon index, the Simpson index [17], and the Dominance index. Sequencing depth was evaluated using Good’s coverage [18], while species evenness was measured using Pielou’s evenness index [19]. 

#### 2.3.6. Beta Diversity

To evaluate community composition complexity and inter-sample (inter-group) differences, beta diversity was computed from weighted and unweighted UniFrac distances in QIIME2. 

Principal coordinate analysis (PCoA) was performed on weighted and unweighted UniFrac distance matrices projected onto orthogonal axes. PCoA was implemented using the ggplot2 package in R (version 4.3.2). Non-metric multidimensional scaling (NMDS) was additionally employed for dimensionality reduction. NMDS was likewise conducted in R using the vegan and ggplot2 packages.

#### 2.3.7. Community Difference Analysis

A series of statistical analyses including *t*-tests, ANOSIM, and LEfSe were applied to characterize microbial community structure. SPSS 19.0 software was used to perform normality and homogeneity of variance tests, followed by independent samples *t*-tests; data not conforming to normal distribution were analyzed using the Mann-Whitney non-parametric test. ANOSIM was performed using Bray-Curtis distance matrices with 900 permutations, implemented via the vegan and ggplot2 packages in R. LEfSe was applied to reveal microbial community characteristics using the LEfSe package in R.

#### 2.3.8. Random Forest Analysis

Random forest analysis was performed to identify the most important microbial taxa using the randomForest package in R. Model construction was implemented at a variable dropout fraction of 0.2, without column-wise normalization, and the top 30 features were preserved. Model performance was evaluated using 10-fold cross-validation, with taxa ranked by MeanDecreaseAccuracy and MeanDecreaseGini.

### 2.4. Untargeted Metabolomics

#### 2.4.1. Metabolite Extraction

Serum samples (100 μL) were transferred to EP tubes, resuspended in pre-chilled 80% methanol, and vortexed thoroughly. Following a 5 min incubation on ice, the samples were centrifuged at 15,000× *g* at 4 °C for 20 min. An aliquot of the resulting supernatant was diluted with LC-MS-grade water to a final methanol concentration of 53%, transferred to a fresh Eppendorf tube, and then subjected to a second centrifugation under identical conditions (15,000× *g*, 4 °C, 20 min). The clarified supernatant was subsequently introduced into the LC-MS/MS system for analysis [20,21].

#### 2.4.2. UHPLC–MS/MS Analyses

UHPLC-MS/MS analyses were conducted by Novogene Co., Ltd. (Beijing, China) on a Vanquish UHPLC system (Thermo Fisher Scientific, Waltham, MA, USA) coupled to either an Orbitrap Q Exactive™ HF or an Orbitrap Q Exactive™ HF-X mass spectrometer (Thermo Fisher Scientific, Waltham, MA, USA). Samples were loaded onto a Hypersil Gold column (100 mm × 2.1 mm, 1.9 μm) and separated over a 12 min linear gradient at a flow rate of 0.2 mL/min. For both positive and negative polarity modes, eluent A consisted of 0.1% FA in water and eluent B consisted of methanol, with the following gradient program: 2% B for 0–1.5 min; 2–85% B over 1.5–3 min; 85–100% B over 3–10 min; 100–2% B at 10.1 min; and re-equilibration at 2% B until 12 min. The Q Exactive™ HF mass spectrometer (Thermo Fisher Scientific, Waltham, MA, USA)was operated in positive/negative polarity mode under the following source parameters: spray voltage 3.5 kV, capillary temperature 320 °C, sheath gas flow rate 35 psi, aux gas flow rate 10 L/min, S-lens RF level 60, and aux gas heater temperature 350 °C.

#### 2.4.3. Data Processing and Metabolite Identification

Raw UHPLC-MS/MS data were processed in Compound Discoverer (version 3.3) for peak alignment, peak picking, and metabolite quantitation. Key parameters were configured as follows: peak areas were corrected against the first QC sample; mass tolerance was set to 5 ppm; signal intensity tolerance was set to 30%; and minimum intensity was applied as a threshold. Peak intensities were subsequently normalized to total spectral intensity, and the normalized data were used to predict molecular formulas based on adduct ions, molecular ion peaks, and fragment ions. Peaks were matched against the mzCloud (https://www.mzcloud.org/, accessed on August 2024), mzVault, and MassList databases to obtain accurate qualitative and relative quantitative results. Statistical analyses were carried out using R (version 4.3.2), Python (version 3.9.7), and CentOS (release 7.9.2009). For non-normally distributed data, values were standardized using the formula sample raw quantitation value(sum of sample metabolite quantitation values/sum of QC1 sample metabolite quantitation values), yielding relative peak areas. Metabolites with a coefficient of variation (CV) of relative peak areas exceeding 30% across QC samples were excluded from further analysis. 

#### 2.4.4. Data Analysis

Metabolite annotation was carried out against the KEGG (https://www.genome.jp/kegg/pathway.html, accessed on August 2024), HMDB (https://hmdb.ca/metabolites), and LIPIDMaps (http://www.lipidmaps.org/, accessed on August 2024) databases. Principal component analysis (PCA) and partial least squares discriminant analysis (PLS-DA) were conducted using metaX [22], a dedicated metabolomics data processing platform. Univariate analysis via t-tests was applied to assess statistical significance, and metabolites meeting the criteria of VIP score > 1, *p*-value < 0.05, and fold change ≥ 1.5 or ≤0.667 were designated as differential metabolites. Volcano plots were generated using the R ggplot2 package (version 3.4.4) to filter metabolites of interest based on log2 (FoldChange) and −log10 (*p*-value). Functional characterization of differential metabolites and their associated metabolic pathways was performed using the KEGG database.

#### 2.4.5. Correlation Analysis

Spearman correlation analysis was applied to examine associations between genera identified through 16S rDNA and differential metabolites from metabolomics, with heatmaps constructed to quantify the degree of association between species diversity and metabolites in environmental samples. O2PLS (orthogonal partial least squares) analysis was performed on the metabolite and microbial datasets using the OmicsPLS package (version 2.0.2) in R, followed by the generation of Top20 metabolite and microbial feature ranking diagrams based on O2PLS importance scores and the corresponding O2PLS loading plots.

*Prevotella_9*, *Treponema*, *Rikenellaceae_RC9_gut_group*, *Oscillospiraceae UCG-005*, *Ruminococcus*, and *Christensenellaceae_R-7_group* were selected for further investigation of their associated metabolites via O2PLS and Mantel test analyses. For O2PLS, the OmicsPLS package in R was used to integrate metabolomics and microbiome datasets, yielding Top20 feature ranking plots and loading plots based on O2PLS importance scores. Mantel test correlation analysis was conducted using the linkET package(version 0.0.7) in R to identify potential associations between the two datasets by evaluating their respective distance matrices. Correlation heatmaps were subsequently generated for the Top20 metabolites and microorganisms, the Top15 metabolites and microorganisms, and the five selected microbial species interacting with the Top20 metabolites.

## 3. Results

### 3.1. Analysis of ASVs

**Figure 1:**
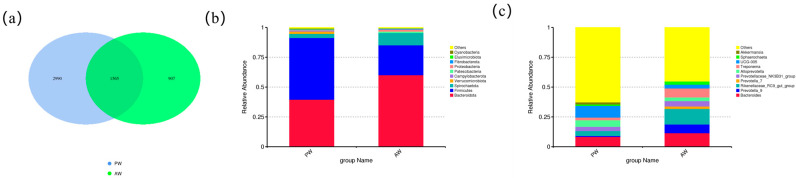
Microbial community composition analysis of Xinggao mutton sheep. (**a**) ASV (Amplicon Sequence Variant)-based Venn diagram. (**b**) Bar plot of the relative abundance of the top 10 species at the phylum level. (**c**) Bar plot of the relative abundance of the top 10 species at the genus level.

A total of 5462 ASVs were identified from the 40 samples via 16S amplicon sequencing (Figure 1a). Of these, 2990 ASVs were unique to the PW group and 907 ASVs were unique to the AW group, while 1565 ASVs were shared between the two groups.

The overall microbial composition at the phylum showed that Bacteroidota, Firmicutes, and Spirochaetota were the most abundant in the two groups (Figure 1b). At the genus level, *Oscillospiraceae UCG-005*, *Bacteroides*, and *Alloprevotella* were the most abundant in the PW group, and *Rikenellaceae_RC9_gut_group*, *Bacteroides*, and *Prevotella_9* were the most abundant in the AW group (Figure 1c).

### 3.2. Analysis of the Diversity of Intestinal Flora

**Figure 2:**
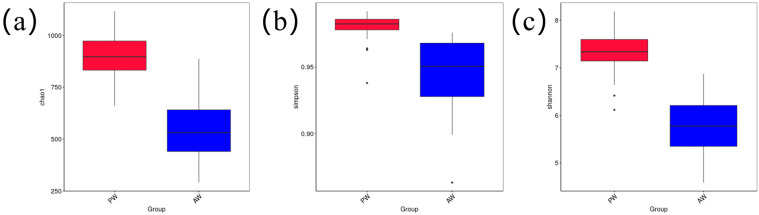
Comparison of alpha diversity indices between groups. (**a**) Box plots of differences in the Chao1 index between groups. (**b**) Box plots of differences in the Simpson index between groups. (**c**) Box plots of differences in the Shannon index between groups.

The α-diversity indices of the intestinal flora in the PW group were all higher than those in the AW group (Figure 2a–c). The Chao1, Simpson, and Shannon indices tended to decrease, suggesting that both the species richness and evenness of the intestinal flora decreased in the AW group compared with the PW group.

**Figure 3:**
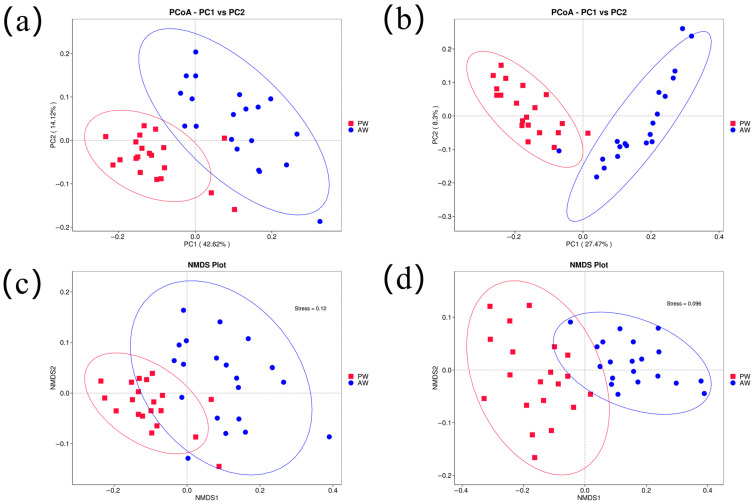
Beta diversity analysis of intestinal microbiota: (**a**,**b**) 2D PCoA plots (**left**: weighted UniFrac distance; **right**: unweighted UniFrac distance); (**c**,**d**) NMDS plots (**left**: weighted UniFrac distance; **right**: unweighted UniFrac distance). The ellipses indicate the 95% confidence interval for each group.

The β-diversity between the two groups was assessed using PCoA based on weighted and unweighted UniFrac distances. Both the weighted and unweighted PCoA showed strong clustering of the microbial communities. In the weighted PCoA, PC1 contributed 42.62% of the variation and PC2 contributed 14.12% of the variation (Figure 3a). In the unweighted PCoA, PC1 contributed 24.7% of the variation and PC2 contributed 8.13% of the variation (Figure 3b). Non-metric multidimensional scaling is a non-linear model based on weighted and unweighted UniFrac distances used to analyze species information in samples. The method was designed to overcome the shortcomings of linear models (including PCA and PCoA) and to better reflect the non-linear structure of ecological data (Figure 3c,d). Additionally, ANOSIM based on Bray-Curtis distance was performed to further evaluate the compositional difference between the two groups, and the results confirmed a significant difference in fecal microbiota structure between the AW and PW groups (*p* < 0.01) (Supplementary Figure S1).

### 3.3. Statistical Analysis

**Figure 4:**
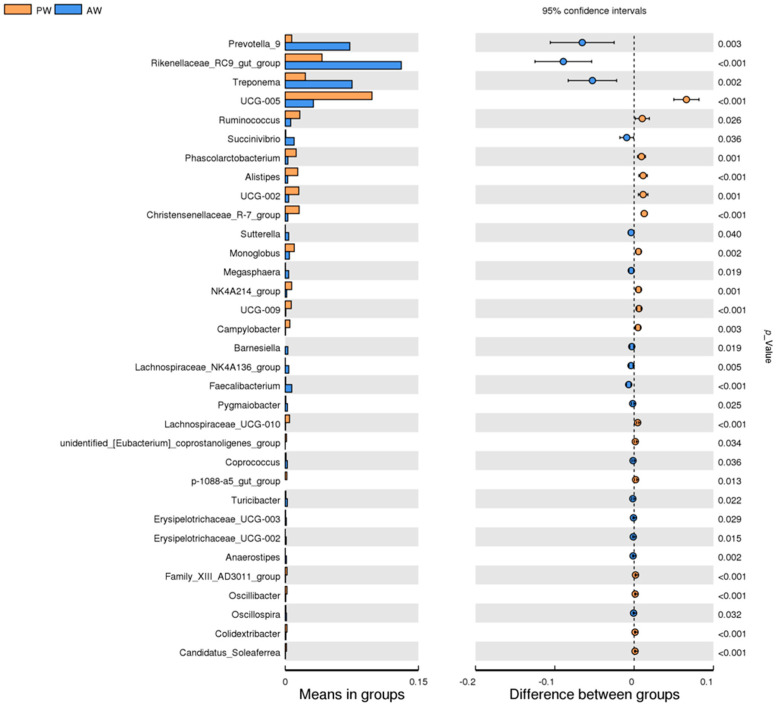
Analysis of species differences between groups.

T-tests were used to identify species that were significantly different between groups at each taxonomic level (*p*-value < 0.05). The results at the phylum and genus levels are shown in Figure 4. At the genus level, the relative abundances of *Prevotella_9* (*p* = 0.0029), *Treponema* (*p* = 0.0017), and *Rikenellaceae_RC9_gut_group* (*p* < 0.0001) were significantly increased in the AW group. The relative abundances of *Oscillospiraceae UCG-005* (*p* < 0.0001), *Ruminococcus* (*p* = 0.0262), and *Oscillospiraceae UCG-002* (*p* = 0.0007) were significantly increased in the PW group.

### 3.4. Species Variability Analysis

**Figure 5:**
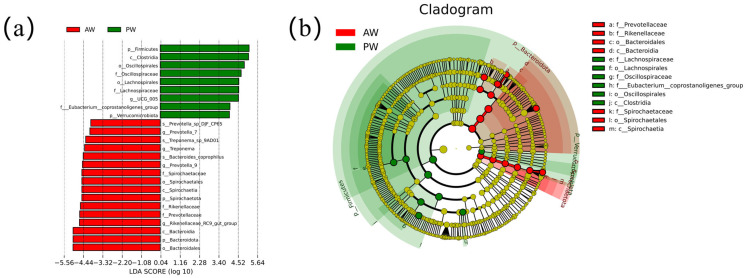
LEfSe analysis of differential intestinal microbiota. (**a**) Evolutionary branching diagram. (**b**) Bar plot of the LDA value distribution.

LEfSe analysis based on a rank test was applied to further determine significantly different abundances between groups. LEfSe analysis differentiated the microbial communities of each group by LDA scores greater than 4. At the phylum level, *Spirochaetota* and *Bacteroidota* were significantly higher in the AW group than in the PW group (Figure 5a,b). At the genus level, *Prevotella_7*, *Prevotella_9*, and *Rikenellaceae* Rcg-gut group were significantly higher in the AW group than in the PW group.

### 3.5. Results of Random Forest Analysis

**Figure 6:**
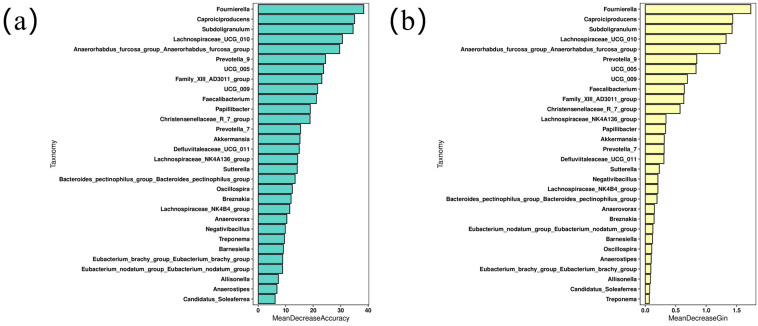
Variable importance analysis. (**a**,**b**) Variable importance ranking chart.

To analyze species abundance using the random forest algorithm, different numbers of species were selected by gradient at various taxonomic levels; random forest models were constructed. Cross-validation (default 10-fold) was carried out for each model, and the most important species were filtered by MeanDecreaseAccuracy and MeanDecreaseGini (Figure 6a,b). Among the top 30 bacterial genera, *Faecalibacterium*, *Butyricicoccaceae UCG-009*, and *Oscillospiraceae UCG-005* were selected as predictors based on their values.

### 3.6. Serum Metabolomics Analysis

**Figure 7:**
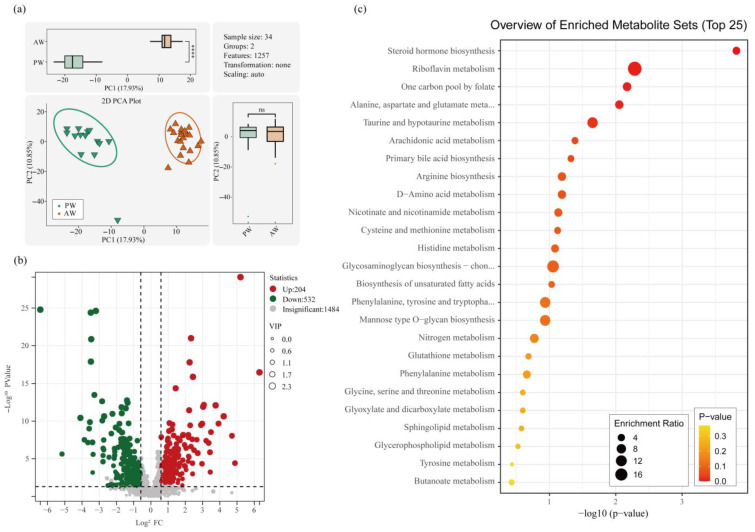
(**a**) Two-dimensional PCA score plot (transformation: none; scaling: UV/auto; sample size: 34; features: 1257), with box plots showing the distribution of PC1 (17.93%) and PC2 (10.85%) scores between the PW and AW groups. **** indicates *p* < 0.0001. (**b**) Volcano map of differential metabolites. (**c**) KEGG enrichment analysis bubble diagram.

The differential metabolites were screened using a non-targeted LC-MS metabolomics assay. Principal component analysis (PCA) revealed a significant difference in PC1 between the PW and AW groups (*p* < 0.001) (Figure 7a). The screening of differential metabolites was based on three parameters: VIP, FC, and *p*-value. VIP refers to the variable importance in the projection of the first principal component of the PLS-DA model [23], and the VIP value indicates the contribution of the metabolite to the grouping. FC refers to the multiplicity of differences (fold change), the ratio of the mean of all biological replicates for each metabolite in the comparison group. *p*-values were calculated using t-tests. Threshold criteria were set as VIP > 1.0, FC > 1.5 or FC < 0.667, and a *p*-value < 0.05 [23,24,25]. There were a total of 736 significantly different metabolites, including 204 metabolites upregulated in the PW group, such as LPS 20:4,2,6-dihydroxypurine, alpha-benzylsuccinic acid, and LPS 18:1, and 532 different metabolites upregulated in the AW group, including (+/−)11-HETE,8-iso-15-keto prostaglandin F2α, 15(R),19(R)-hydroxy prostaglandin F1α, and tyrosol (Figure 7b).

Differential metabolites were clustered using correlation pathway analyses to identify the metabolic pathways significantly altered under the experimental conditions. The results are presented in a bubble diagram in Figure 7c. The significantly altered metabolic pathways included taurine and hypotaurine metabolism, cysteine and methionine metabolism, and steroid hormone biosynthesis.

### 3.7. Correlation Analysis of Gut Microbiota with Metabolites

**Figure 8:**
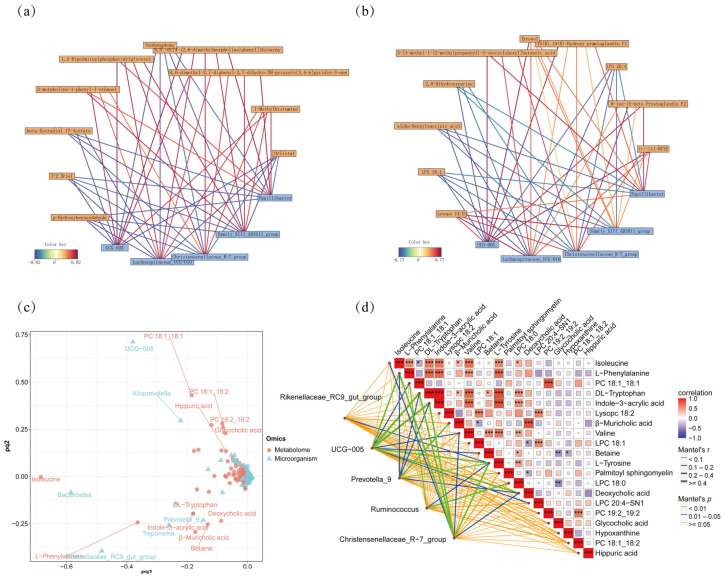
Correlation analysis of metabolome and microbiome. (**a**) Correlation network diagram in positive ion mode. (**b**) Correlation network diagram in negative ion mode. (**c**) O2PLS loading plot. (**d**) Mantel test correlation heatmap. * indicates *p* < 0.05, ** indicates *p* < 0.01, *** indicates *p* < 0.001.

Spearman correlation analysis was performed between differential flora and differential metabolites (Figure 8a,b). The results showed that *Oscillospiraceae UCG-005* was positively correlated with 3-[4-methyl-1-(2-methylpropanoyl)-3-oxocyclohexyl] butanoic acid, 11-HETE, tyrosol, 15(R),19(R)-hydroxy prostaglandin F1α, lysope 14:0, and 8-iso-15-keto prostaglandin F2α, and was negatively correlated with alpha-benzylsuccinic acid, 2,6-dihydroxypurine, LPS 20:4, and LPS 18:1. *Christensenellaceae_R-7_group* was positively correlated with 8-iso-15-keto prostaglandin F2α, 3-[4-methyl-1-(2-methylpropanoyl)-3-oxocyclohexyl] butanoic acid, 15(R),19(R)-hydroxy prostaglandin F1α, tyrosol, 11-HETE, and lysope 14:0, and was negatively correlated with alpha-benzylsuccinic acid, 2,6-dihydroxypurine, LPS 20:4, and LPS 18:1. *Papillibacter* was positively correlated with lysope 14:0, 8-iso-15-keto prostaglandin F2α, 3-[4-methyl-1-(2-methylpropanoyl)-3-oxocyclohexyl]butanoic acid, 11-HETE, and 15(R), 19(R)-hydroxy prostaglandin F1α, and was negatively correlated with 2,6-dihydroxypurine, LPS 20:4, LPS 18:1, and alpha-benzylsuccinic acid. *Lachnospiraceae_UCG-010* was positively correlated with 3-[4-methyl-1-(2-methylpropanoyl)-3-oxocyclohexyl] butanoic acid, lysope 14:0, (+/−)11-HETE, tyrosol, 8-iso-15-keto prostaglandin F2α, and 15(R), 19(R)-hydroxy prostaglandin F1α, and was negatively correlated with alpha-benzylsuccinic acid, LPS 18:1, LPS 20:4, and 2,6-dihydroxypurine. *Family_XIII_AD3011_group* was positively correlated with lysope 14:0, 3-[4-methyl-1-(2-methylpropanoyl)-3-oxocyclohexyl] butanoic acid, 11-HETE, tyrosol, 15(R), 19(R)-hydroxy prostaglandin F1α, and 8-iso-15-keto prostaglandin F2α, and was negatively correlated with alpha-benzylsuccinic acid, LPS 18:1, LPS 20:4, and 2,6-dihydroxypurine. The O2PLS loadings plot demonstrates the integrated analysis of the microbiome and metabolome (Figure 8c). The figure labels multiple microbial genera (*Alloprevotella*, *UCG-005*) and metabolites (hippuric acid, glycocholic acid, and PC 18:1_18:1), where their positions reflect their contributions to the principal component. The distribution pattern reveals correlations between microorganisms and metabolites, suggesting that microbial community structure is associated with host metabolic states, though causal directionality cannot be determined from correlational analyses alone.

The Mantel test showed that *Prevotella_9*, *Treponema*, *Rikenellaceae_RC9_gut_group*, *Oscillospiraceae UCG-005*, *Ruminococcus*, and *Christensenellaceae_R-7_group* were significantly associated with the metabolome (Figure 8d). *Rikenellaceae_RC9_gut_group* was positively correlated with L-tyrosine. *UCG-005* was positively correlated with L-tyrosine, Indole-3-acrylic acid, and PC 18:1_18:1. *Prevotella_9* was positively correlated with L-tyrosine, Indole-3-acrylic acid, and L-Phenylalanine. *Ruminococcus* was positively correlated with L-tyrosine, PC 18:1_18:1, and LPC 18:0. *Christensenellaceae_R-7_group* was positively correlated with L-tyrosine, Indole-3-acrylic acid, and L-Phenylalanine. It should be noted that these correlations reflect statistical associations only; the directionality and causality of the relationships between gut microbiota and metabolites require further validation through mechanistic or interventional studies.

## 4. Discussion

The gastrointestinal environment of newborn lambs is highly unstable, and their immune system and physiology undergo development and maturation processes. Their intestinal flora is highly influenced by age, diet, environment, and subsequent weaning stress. The time-related dynamics of the microbial communities in the jejunum and colon of lambs from the immature stage to the ruminant stage were studied. *Bacteroides* was the most abundant bacterial phylum during the growth and development of lambs from the day of birth to the 84th day, and its relative abundance decreased with age, whereas the relative abundance of *Actinobacteria* increased with age. In our study, *Oscillospiraceae UCG-005*, *Ruminococcus*, and *Oscillospiraceae UCG-002* were the dominant bacteria at the genus level in the pre-weaning group. *Ruminococcus* harbors genes encoding various peptidases. *Clostridium* and *Lactobacillus* contain branched-chain amino acid aminotransferases [26]. *Ruminococcus* participates in the decomposition of cellulose and starch. Members of this genus secrete abundant propionic and acetic acids [27]. *Ruminococcus* is among the most efficient bacterial genera for carbohydrate catabolism. Some of the *Ruminococcus* bacteria obtain nutrients by degrading the cellulose in the host’s digestive system [28]. In the rumen of buffalo, the relative abundance of *Ruminococcus* sp. decreases as the animal ages, suggesting that other microorganisms play additional roles in carbohydrate and cellulose degradation [29]. The decline in *Ruminococcus* post-weaning in our Xinggao sheep is consistent with cross-species findings. In foals, *Ruminococcus* abundance decreased significantly within 3 days post-weaning regardless of weaning method [30]. Similarly, in dairy calves, *Ruminococcus* declined at weaning irrespective of weaning age [31]. In piglets, early weaning reduced colonic *Ruminococcus*, which was positively correlated with intestinal barrier integrity [32]. These observations suggest that the weaning-associated reduction in *Ruminococcus* in Xinggao sheep reflects a conserved gut microbial response to dietary and stress challenges during the weaning transition.

At the genus level, *Prevotella_9*, *Treponema*, *Rikenellaceae_RC9_gut_group*, and *Bacteroides* were dominant in the AW group. Post-weaning, plant glycan fermenters (e.g., Prevotella-9) seemed to replace milk-glycan-fermenting *Fusobacterium* and *Bacteroides*. Post-weaning, *Prevotella-9* became dominant [33]. Small amounts of plant glycans, such as starch in the digesta, may have supported the growth of starch-degrading taxa, including *Turicibacter*, *Terrisporobacter*, *Porphyromonas*, and *Prevotella* [34,35,36]. The post-weaning increase in *Prevotella_9* observed in Xinggao sheep is consistent with findings across multiple weaning studies in pigs. In a field study of commercial piglets, *Prevotella* abundance was significantly higher in healthy weaned animals compared to diarrheic ones, suggesting its role in gut homeostasis during dietary transition [37]. A longitudinal metagenomic study comparing weaning at 14, 21, and 28 days of age reported that multiple *Prevotella* species—including *P. copri*, *P. pectinovora*, and *P. stercorea* increased in relative abundance immediately post-weaning regardless of weaning age, alongside other SCFA-producing taxa, reflecting the microbial adaptation to plant-based solid feed [38]. In a large longitudinal cohort study, *Prevotella_9* specifically proliferated after weaning and was positively associated with average daily gain; faster microbiota maturation, including *Prevotella* enrichment, was typical of animals with superior growth performance [39]. Taken together, these findings support the interpretation that the post-weaning increase in *Prevotella_9* in Xinggao sheep reflects an adaptive microbial response to the shift from milk- to plant-based nutrition, rather than a species-specific phenomenon.

The rumen bacteria *Treponema* degrade plant polysaccharides, such as xylan, pectin, and arabinogalactan, and produce volatile fatty acids, which serve as the major energy source for ruminants. Some strains of *Treponema*, such as T. bryanti, have been shown to interact with the cellulolytic bacterium *F. succinogenes*. The genus *Treponema*, a member of the phylum *Spirochetes*, is always detected in the gastrointestinal tracts of ruminants; these bacteria contain a wide variety of carbohydrate-active enzymes, as demonstrated via genome analysis [40], and are reported to be associated with the degradation of cellulose and pectin [41]. Consistent with the enrichment of *Treponema* observed in the present study following weaning in Xinggao sheep, similar patterns have been reported in other livestock during the weaning transition. In Bama miniature pigs, *Treponema* abundance increased significantly after weaning, coinciding with a dietary shift from milk to solid plant-based feed, suggesting its role in adapting to complex carbohydrate digestion [42]. Across a broader developmental timeline in commercial pigs, *Treponema* was identified as a growing-stage-associated genus and a key constituent of the *Prevotella*-*Lactobacillus*-*Treponema* enterotype, which became predominant in older animals [43]. These findings collectively suggest that the post-weaning enrichment of *Treponema* in Xinggao sheep may reflect a conserved microbial adaptation to increased dietary fiber intake, reinforcing its functional relevance in plant polysaccharide degradation during this critical nutritional transition.

In the present study, the relative abundance of *Rikenellaceae_RC9_gut_group* was significantly increased post-weaning in Xing lambs, yet the level of acetoacetate—a direct product of butyrate β-oxidation—was significantly decreased, suggesting that weaning stress may impair the capacity of intestinal epithelial cells to oxidize and utilize butyrate, rather than affecting its microbial production. In contrast, previous studies in other animals have reported opposite findings. Wang et al. demonstrated a significant positive correlation between *Rikenellaceae RC9 gut group* abundance and colonic acetate and butyrate concentrations in mice [44], and analogous associations between *RC9* abundance and volatile fatty acid levels have also been reported in ruminants, such as Tarim wapiti [45]. However, this discrepancy may be attributed to differences in dietary fiber composition and gastrointestinal physicochemical conditions across host species, which may modulate the fermentation efficiency and metabolic output of the *RC9 gut group* independently of its relative abundance.

*Bacteroides* species are predominantly commensal colonizers of the adult gut and are involved in resistance to enteric pathogens [46]. However, conflicting evidence from fecal and metagenomics-derived investigations has demonstrated the complex and multifaceted roles of *Bacteroides* strains in intestinal infections. A previous study [47] found that the microbiota were dominated by the phyla *Firmicutes*, *Bacteroides*, and *Proteobacteria*. During the early-life stages, the genera *Bacteroides*, *Escherichia/Shigella*, and *Clostridium* cluster XIVa were abundant during the preweaning period, while *Prevotella* dominated post-weaning. Saladrigas-García et al. [48] identified early intestinal colonizers as belonging to the genera *Bacteroides*, Escherichia-Shigella, *Clostridium* sensu stricto 1, and *Fusobacterium*. During lactation (days 7 to 21), the higher relative abundances of *Bacteroides* and *Lactobacillus* are correlated with a milk-oriented microbiome [49]. *Bacteroides* has been reported to use a wide range of both milk oligosaccharides and host-derived glycans [50]. Lactobacillus is a well-known lactate producer [51]. Kazachstania slooffae has been linked to the provision of amino acids and energy to bacteria such as *Lactobacillus* and *Prevotella*, as well as the host piglets [52,53].

In our study, the significantly altered metabolic pathways included taurine and hypotaurine metabolism, cysteine and methionine metabolism, and steroid hormone biosynthesis. Comparing the PW group with the post-weaning AW group in Xinggao mutton sheep, we observed pronounced perturbations in sulfur amino acid metabolism that mirrored the nutritional and oxidative challenges imposed by the weaning transition. Cysteine is a rate-limiting substrate for the synthesis of glutathione (GSH) and taurine, metabolites that play pivotal roles in cellular redox status and osmoregulation [54]. Consistent with our findings, a multi-omics study in Bama miniature pigs identified taurine and hypotaurine metabolism as one of the five KEGG pathways significantly downregulated following weaning. The authors attributed this suppression to the heightened inflammatory stress associated with the dietary transition, as the demand for taurine-related antioxidant compounds increases while their synthesis capacity may be impaired under acute stress conditions [42]. The parallel suppression of this pathway across divergent species suggests a conserved metabolic vulnerability in the taurine biosynthesis axis during weaning-induced oxidative stress. In newly weaned pigs, systemic cysteine is not effectively utilized for gut GSH production; instead, it is oxidized to taurine and eliminated in the bile [55], indicating that both the cysteine flux and its downstream taurine output are jointly dysregulated in the peri-weaning period. In the AW group of Xinggao lambs, the concurrent alteration of cysteine and methionine metabolism further supports the interpretation that sulfur amino acid homeostasis is comprehensively disrupted following weaning. Zhou found that dietary taurine supplementation (2–3%) could enhance growth performance, reduce diarrhea rates, ameliorate oxidative stress and inflammation, and promote intestinal barrier function in weaned piglets [56]. We therefore speculate that in Xinggao mutton sheep, the weaning-induced depression of taurine and hypotaurine metabolism represents an endogenous antioxidant deficit specific to the milk-to-solid feed transition, and that targeted taurine supplementation during the peri-weaning window may constitute a breed-specific nutritional strategy to alleviate weaning stress and support gut health in this newly cultivated breed.

Our results showed that *Oscillospiraceae UCG-005* was positively correlated with 3-[4-methyl-1-(2-methylpropanoyl)-3-oxocyclohexyl] butanoic acid, 11-HETE, tyrosol, 15(R),19(R)-hydroxy prostaglandin F1α, LysoPE 14:0, and 8-iso-15-keto prostaglandin F2α. Given that *UCG-005* was the dominant genus in the PW group but declined markedly after weaning, its positive correlation with these metabolites suggests that the weaning-induced reduction in *UCG-005* may contribute to the concurrent perturbation of lipid mediator and antioxidant metabolite profiles in Xinggao sheep. Fecal microbiota transplantation studies in weaned piglets have demonstrated that promoting *UCG-005* colonization alleviates oxidative stress and supports intestinal barrier integrity [57]. Among the correlated metabolites, tyrosol is a phenolic compound endogenously produced by gut microbiota through microbial metabolism of tyrosine; preclinical studies have consistently linked tyrosol to potent anti-inflammatory effects and protective roles against gastrointestinal disorders, including ulcerative colitis and colorectal cancer [58]. The metabolites 11-HETE, 15(R),19(R)-hydroxy prostaglandin F1α, and 8-iso-15-keto prostaglandin F2α are derivatives of the arachidonic acid cascade; gut microbiota enzymes can modulate the circulation of eicosanoids derived from arachidonic acid metabolism, thereby influencing the initiation or resolution of inflammation in the host [59]. LysoPE 14:0, a bioactive lysophospholipid, participates in membrane signaling and immune regulation. Collectively, the positive correlations between *UCG-005* and these metabolites suggest that this genus may play a role in maintaining antioxidant capacity and modulating inflammatory lipid signaling during the weaning transition in Xinggao mutton sheep, and its decline post-weaning may represent one microbial mechanism underlying weaning-associated intestinal stress.

## 5. Conclusions

By integrating 16S rRNA amplicon sequencing with untargeted metabolomics, we delineated the gut microbiota-metabolome axis underlying the weaning transition in Xinggao sheep. Weaning drove a phylum-level shift from *Firmicutes* to *Bacteroidota* dominance alongside reduced microbial diversity, with concomitant perturbations in taurine/hypotaurine and cysteine/methionine metabolism. Critically, *Oscillospiraceae UCG-005* and *Ruminococcus*, which declined markedly post-weaning, were strongly correlated with L-tyrosine, Indole-3-acrylic acid, and LysoPE 14:0-metabolites with established roles in intestinal immune homeostasis and epithelial integrity—identifying them as prioritized probiotic candidates for peri-weaning intervention. In parallel, the pronounced upregulation of taurine metabolism provides a mechanistic rationale for dietary taurine supplementation to attenuate weaning-induced oxidative stress and diarrhea. More broadly, L-tyrosine, Indole-3-acrylic acid, and LysoPE 14:0 are proposed as accessible serum biomarkers for early gut health surveillance in neonatal ruminants. These findings establish a multi-omics framework that moves beyond descriptive microbial profiling toward translatable strategies for improving lamb welfare and production resilience during the critical weaning window.

## Data Availability

The original contributions presented in the study are included in the Article/Supplementary Material; further inquiries can be directed to the corresponding author.

## Supplementary Materials

The following supporting information can be downloaded at: https://www.mdpi.com/article/10.3390/ani16101532/s1.
