# Supplementary figures and images for: Dynamic Shifts in Gut Microbiota and Metabolic Pathways of Xinggao Mutton Sheep During Weaning: A Multi-Omics Analysis
Source: Animals (Basel). 2026 May 16;16(10):1532. doi: 10.3390/ani16101532 (PMC13203436; doi:10.3390/ani16101532)

# Bray-Curtis Anosim

R=0.8898; p=0.0011

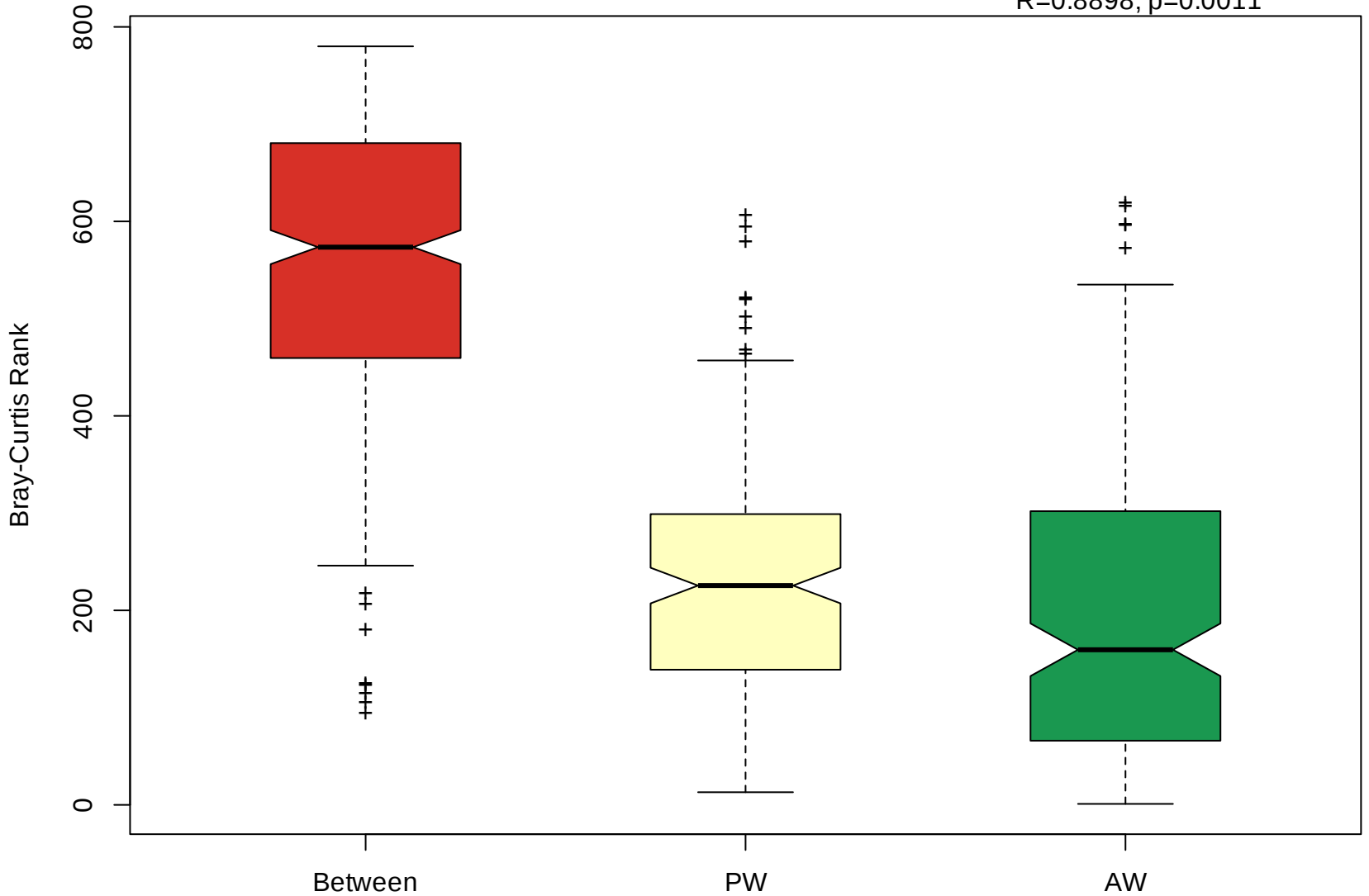

Supplement: Supplementary file 1 [file animals-16-01532-s001.zip › Supplementary Figure S1.pdf]
